# Interplay of Carbon Content and Sintering Temperature on Microstructure and Mechanical Behavior in Ultrafine-Grained WC-10CoNiFe and WC-10Co Cemented Carbides

**DOI:** 10.3390/ma18122789

**Published:** 2025-06-13

**Authors:** Ji Zhang, Kun Li, Yubo Chen, Cheng Qian, Shuailong Zhang, Huichao Cheng

**Affiliations:** 1 State Key Laboratory of Powder Metallurgy, Central South University, Changsha 410083, China; 223312142@csu.edu.cn (J.Z.); kunlee@csu.edu.cn (K.L.); 22024088@csu.edu.cn (C.Q.); 223312139@csu.edu.cn (S.Z.); 2 Jiangxi Tungsten Advanced Materials Innovation Research Co., Ltd., Nanchang 330096, China

**Keywords:** cemented carbide, sintering temperature, carbon content, grain size, mechanical properties

## Abstract

As a crucial component of cemented carbide, the binder phase exerts a profound influence on its microstructure and mechanical properties. In this study, ultrafine-grained WC-10CoNiFe and WC-10Co cemented carbides, with grain sizes ranging from 0.25 to 0.4 μm, were fabricated via powder mixing, forming, and sintering processes utilizing 0.4 μm WC powder as the starting material. The effects of carbon content (5.44–5.50 wt%) and sintering temperatures (1410–1500 °C) on the grain organization and mechanical properties of these cemented carbides were systematically investigated. The results revealed that WC-10CoNiFe achieved its optimal mechanical properties at a carbon content of 5.46 wt% and a sintering temperature of 1450 °C, exhibiting a flexural strength of 2999 MPa and a hardness of 1765 HV. Likewise, WC-10Co attained its peak performance at a carbon content of 5.48 wt% and a sintering temperature of 1410 °C, with a flexural strength of 3598 MPa and a hardness of 1853 HV. Remarkably, the finer grain size of the WC-10CoNiFe alloy (0.261 µm), compared to that of WC-10Co (0.294 µm), can be ascribed to the suppression of the dissolution–reprecipitation process by the multi-principal-element alloy binder. This study demonstrated the synergistic regulation of microstructure and mechanical properties in ultrafine-grained cemented carbides through the incorporation of a multi-principal-element alloy binder. This innovative strategy not only effectively refines the grain size but also endows the alloy with exceptional mechanical properties, offering a valuable new perspective for the research and development of high-performance cemented carbides.

## 1. Introduction

Ultrafine cemented carbide, a composite material prepared via the powder metallurgy method, comprises refractory metal compounds (WC, TaC, NbC, etc.) with a particle size of 0.2–0.5 μm as the matrix and soft ductile metals (Co, Ni, Fe, etc.) as the binder phase [1,2,3,4]. This material is characterized by high strength, high hardness, a high modulus of elasticity, high compressive strength, wear resistance, a low coefficient of thermal expansion, and excellent oxidation and corrosion resistance. These exceptional properties render ultrafine cemented carbides indispensable for precision machining applications and operations in extreme environments, leading to their widespread utilization in various fields such as broaching tools, molds, impact tools, and wear- and corrosion-resistant parts [5,6,7,8].

Currently, WC-Co cemented carbides constitute the majority of ultrafine crystalline cemented carbides employed in practical production, and research in this area has been extensive. For instance, Gao et al. [9] utilized oscillatory sintering and forging (OSF) technology to fabricate ultrafine crystalline WC-Co cemented carbide, achieving a hardness of 1911 kg/mm^2^ and a fracture toughness of 17.52 MPa·m^1/2^ at 1125 °C. This represents a 60 kg/mm^2^ increase in hardness compared to conventional methods, demonstrating a synergistic optimization of hardness and toughness. Liu et al. [10] prepared ultrafine crystalline WC-Co cemented carbide by doping yttrium in ammonium paratungstate solution, resulting in a tungsten carbide powder refined to 0.32 μm. The alloy exhibited a hardness of 94.6 HRA and a flexural strength (TRS) of 4841 MPa, with finer tungsten carbide grains (0.28 μm) and a more homogeneous microstructure than those produced by conventional methods. Li et al. [11] achieved a synergistic optimization of hardness, strength, and toughness by optimizing Cr_2_(C,N) addition and WC particle size in ultrafine WC-Co cemented carbide. With 0.5 wt% Cr_2_(C,N) and 0.4 μm WC powders, the alloy attained a flexural strength of 4748 MPa, a hardness of 1786 kg/mm^2^, and a fracture toughness of 10.1 MPa·m^1/2^.

Despite the excellent mechanical properties of ultrafine-grained WC-Co cemented carbides, their performance is significantly reliant on cobalt resources. As a strategic material, cobalt is not only scarce and costly but also poses toxicity and pollution concerns that cannot be overlooked [12]. Therefore, exploring alternative solutions to replace or partially substitute the Co binder phase is of paramount importance for the production and application of ultrafine-grained cemented carbides. Shichalin et al.’s [13] research indicates that WC-based cemented carbides prepared using different binders (Co, Fe, Ni, Cr, Ti) in conjunction with Spark Plasma Sintering (SPS) technology exhibit significant differences in their characteristics and properties. Specifically, alloys utilizing Co as the binder demonstrate the highest density and most uniform binder distribution. In contrast, Fe and Ni binders exhibit uneven distribution and contain residual pores. When Cr and Ti are employed as high-melting-point binders, the sintering process proceeds through a reactive synthesis mechanism accompanied by phase transformations, resulting in a decrease in the WC volume fraction to 47%, thereby significantly impacting the alloy’s hardness, fracture toughness, and strength. Furthermore, the Ti binder promotes the formation of the W2C phase within the alloy, while the Cr binder leads to the formation of hard and brittle chromium carbides, both of which further influence the alloy’s mechanical properties. This study provides crucial insights for the application of multi-principal-element binders in WC-based cemented carbides. Buravlev et al.’s [14] research demonstrates that a WC-4 wt%–TiC-3 wt%–TaC-12 wt% Co composite ceramic wear-resistant alloy, prepared via spark plasma sintering following mechanical activation pretreatment, achieves optimal structural uniformity, density (99.2% TD), and physic mechanical properties (hardness: 1820 HV, fracture toughness: 12.5 MPa·m^1/2^) at 1200 °C. The mechanical activation process aids in breaking down agglomerates and forming a unimodal particle-size distribution, thereby facilitating densification during subsequent SPS processes. The alloy’s microstructure comprises a refractory skeleton composed of WC grains, along with uniformly distributed TiC and TaC carbide particles throughout the entire volume. The excellent fluidity of the cobalt binder contributes to the formation of a dense and low-porosity material. Samples sintered at 1200 °C exhibit high physicomechanical properties, including a relative density of 99.99%, a hardness of HV30 1623.2, a flexural strength of 1125.1 MPa, and a fracture toughness of 10.5 MN·m^−1/2^. Additionally, preliminary wear-resistance evaluations indicate that this material demonstrates good durability in cutting operations, suggesting its potential as a cutting tool material. This study highlights the advantages of combining multi-principal-element binders with mechanical activation pretreatment in enhancing alloy performance.

Multi-principal-element alloys (MPEAs), defined as alloys containing at least four metallic elements with each element comprising 5–35% of the atomic percentage [15,16], have gained attention in recent years due to their high strength, toughness, and corrosion resistance. These properties make MPEAs promising candidates as binder phases for cemented carbides.

Qian et al. [17,18] demonstrated the potential of MPEAs by using CoNiFeCr MPEA as the binder phase in functional gradient cemented carbide (FGCC). The resulting material exhibited a wear rate of 6.95 × 10^−6^ mm^3^/(N m) at 600 °C, which was lower than that of conventional non-gradient cemented carbide (CC) (1.46 × 10^−5^ mm^3^/(N m)). Additionally, the hardness of the FGCC reached 1233 HV, showcasing excellent wear resistance, oxidation resistance, and fracture toughness. Liu et al. [19] prepared WC-based cemented carbide using CoCrNi MPEA as the binder and observed a significant reduction in corrosion current density by approximately 70% and an increase in polarization resistance by one order of magnitude in simulated drilling fluids (pH = 10). The material also exhibited a hardness of 1489.6 HV, highlighting its superior corrosion resistance and mechanical properties. Chen et al. [20] utilized CoCrFeNiCu MPEA as the binder phase in WC-10 wt% HEA composites prepared by microwave sintering. The composites featured a WC grain size of 273 ± 6 nm when sintered for 10 min at 1400 °C, which was 38% smaller than that of WC-Co carbide. Furthermore, the hardness reached 17.6 ± 0.8 GPa, and the fracture toughness was 8.7 ± 0.3 MPa·m^1/2^, demonstrating outstanding mechanical properties and high-temperature stability. These findings collectively suggest that MPEAs are an ideal binder-phase composition system with the potential to replace Co in cemented carbides [17,21].

There exists a close correlation between the organizational properties of cemented carbide and the carbon content and sintering temperature. During the sintering process, the carbon content plays a dual role. On the one hand, it regulates the solubility of tungsten in the binder phase; on the other hand, it effectively inhibits the formation of the deleterious η-phase, thereby safeguarding the overall properties of the alloy [22]. Meanwhile, an increase in sintering temperature significantly enhances the densification of cemented carbide, which is one of the key factors to improve the performance of the alloy. However, excessively high sintering temperatures may trigger unfavorable phenomena such as abnormal grain growth and dimensional deformation, which may, on the contrary, cause damage to the alloy properties [23,24].

In this paper, we innovatively introduced the multi-principal-element alloy binder phase into the ultrafine crystal cemented carbide system and proposed to construct a multi-principal-element alloy binder-phase ultrafine cemented carbide system. The study aims to integrate the high strength, high hardness, and excellent wear resistance of the ultrafine grain structure with the unique advantages of the multi-principal-element alloy binder phase to replace the traditional cobalt-bonding phase. The synergistic effect of multiple factors, such as binder-phase composition, carbon content, and sintering temperature, is investigated systematically to reveal its regulatory mechanism and influence on the mechanical properties of ultrafine-grained cemented carbide.

## 2. Materials and Methods

### 2.1. Materials

The experimental materials were selected from tungsten carbide powder (particle size 0.4 μm, carbon content 6.01 wt%), cobalt powder (1.33 μm), nickel powder (2.0 μm), iron powder (3.08 μm), carbon black (11.8 μm), and ultrafine grain growth inhibitors Cr_3_C_2_ (2.59 μm) and VC (1.94 μm). Two alloy systems were designed for the study: Group A (WC-10Co) with a conventional cobalt binder and Group B (WC-10CoNiFe) with a multi-principal-element alloy binder. To address the insufficient carbon content of the pristine WC powder (a theoretical carbon value of 6.11 wt%), the total carbon content of the alloy was adjusted by the addition of carbon black, resulting in four carbon contents of 5.44, 5.46, 5.48, and 5.50 wt%. Subsequently, eight powder mixtures (A1–A4 and B1–B4) were prepared. Each group of powder mixtures was sintered at three temperatures: 1410 °C, 1450 °C, and 1500 °C, respectively, yielding a total of 24 specimens.

**Figure 1:**
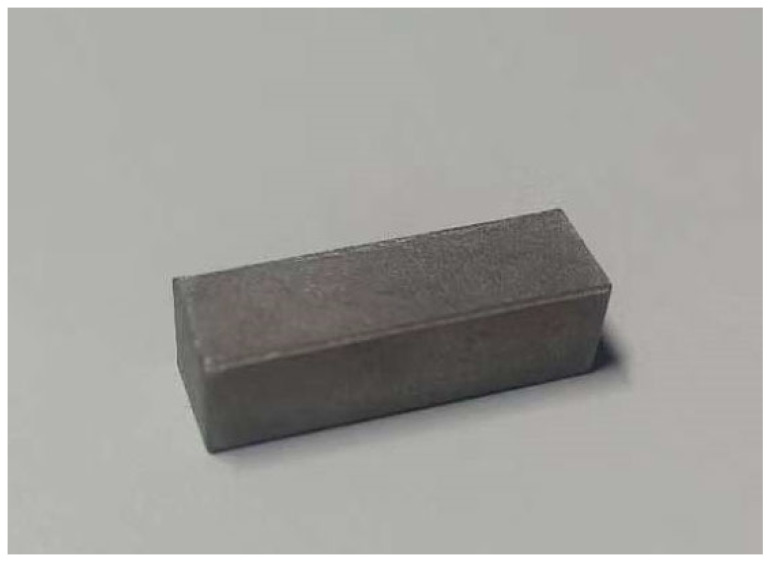
The bending bars for the experiment.

**Figure 2:**
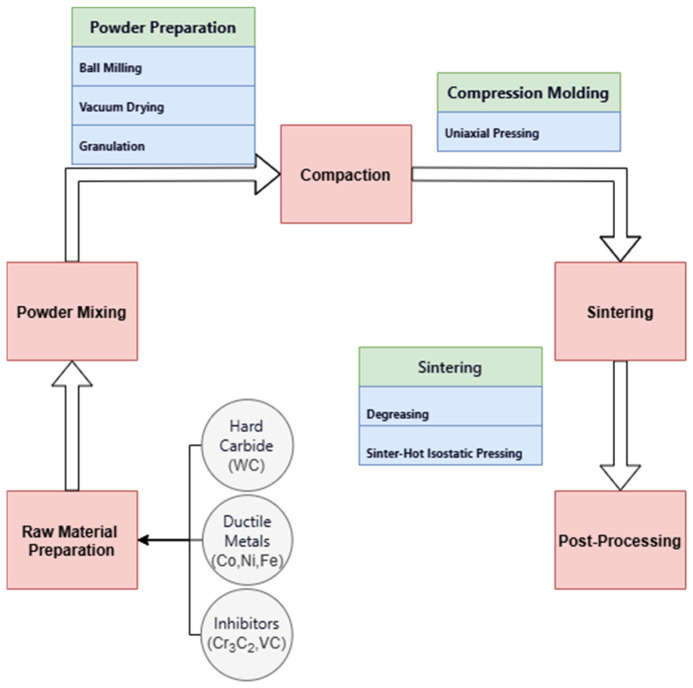
Preparation process of ultrafine-grained multi-principal-element binder-phase cemented carbide.

As detailed in Table 1, the experiments were conducted using a planetary ball mill at 420 r/min for 60 h of mixing and wet grinding to ensure that the powder ingredients were fully refined and uniformly mixed. The resulting slurry was dried in a vacuum drying oven for 24 h, followed by sieving and granulation to optimize powder flow. The forming process was carried out by unidirectional pressing, where the powder was pressed through a mold to fabricate a compact. At the sintering stage, the briquettes were placed in a low-pressure sintering furnace under an argon gas-protected atmosphere at three temperatures: 1410 °C, 1450 °C, and 1500 °C. The argon gas pressure was controlled at 5.0 MPa. The final standard bending-bar samples, as illustrated in Figure 1, were obtained as shown in Table 2, with dimensions of approximately 20 × 6.5 × 5.25 mm. The preparation process is illustrated in Figure 2.

### 2.2. Methods

The samples were subjected to a comprehensive analysis focusing on their microstructure and mechanical properties, with the parameters of each experimental equipment detailed in Table 3.

(1) X-ray diffraction analysis

The specimens were individually examined using a D/max 2550 fully automated rotary-target X-ray diffractometer (D/MAX-2250, Rigaku, Tokyo, Japan). The analysis was performed with Cu target Kα radiation, scanning over a 2θ range from 20° to 80°, at a scanning speed of 5 deg/min and a step size of 0.02 deg. The acquired X-ray diffraction data were processed using Jade 6.5 software to determine the phase composition, crystalline index, and other relevant information of the specimens.

(2) Scanning electron microscopy analysis

The microstructure of the samples and the fracture morphology resulting from the flexural strength test were analyzed using a Quanta FEG250 field-emission scanning electron microscope (Quanta FEG250, FEI, Hillsboro, OR, USA). Additionally, the cemented carbide microstructure was examined with an energy spectrometer (EDS) integrated into the electron microscope. The obtained grain images were quantitatively analyzed using ImageJ 1.8.0 software, applying the linear intercept method. This method involves drawing one or more straight lines on metallographic micrographs and counting the number of intersections between these lines and grain boundaries to calculate the average grain size. The average interception length is determined using the formula:

(1) $$L=\frac{L_{T}}{P\times M}$$

where *L_T_* is the total length of the intercept line, *P* is the number of intersections, and *M* is the magnification of the micrograph.

(3) Microhardness testing

The hardness of the samples was evaluated using the HVS-5 microhardness testing system (BUEHLER5104, Buehler, Lake Bluff, IL, USA). A meticulously ground and polished rectangular specimen was selected, and a tetragonal cone-shaped diamond indenter was pressed into the polished upper surface under a load of 10 kgf. The resulting rhombic indentation was observed under an optical microscope, and the diagonal length of the indentation was measured. Ten points were measured per specimen, and the Vickers hardness was calculated according to Equation (2), with the results averaged arithmetically.

(2) $$\mathrm{HV}=\frac{2F\sin136^{\circ }}{2d^{2}}=\frac{1.854F}{d^{2}}$$

In the equation, *F* represents the applied load, which is 98.07 N, and *d* denotes the mean diagonal length of the indentation, measured in millimeters.

(4) Transverse rupture strength testing

Unlike materials commonly assessed through tensile and compression tests for strength and toughness, cemented carbides, being brittle, are challenging to test in tension. Bending tests, which do not produce significant plastic deformation and easily yield the maximum bending stress, are typically employed to evaluate the strength and toughness of cemented carbides in industrial settings.

In this study, the flexural strength of the specimens was tested using a dynamic and static electro-hydraulic servo testing machine (JXA-8230, JEOL, Tokyo, Japan) according to the ISO 3327-2009 standard, employing the three-point bending method. The flexural strength value was calculated using Equation (3):

(3) $$R=\frac{3Fl}{2bh^{2}}$$

where *R* represents the flexural strength in MPa, *F* is the force required to fracture the specimen in N, *l* is the distance between the two support points in mm, *h* is the height of the specimen in mm, and *b* is the width of the specimen in mm. The specimens used in this test were B type, with dimensions of 20 × 6.5 × 5.25 mm and a span of 14.5 mm. Three specimens of each type were tested, and the average value was taken as the final flexural strength.

**Table 3:** Parameters of experimental equipment.

Installations	Model Number	Company
Scanning electron microscope	Quanta FEG250	FEI, Hillsboro, OR, USA
Microhardness testing system	BUEHLER5104	Buehler, Lake Bluff, IL, USA
X-ray diffractometer	D/MAX-2250	Rigaku, Tokyo, Japan
Dynamic and static electro-hydraulic servo testing machine	JXA-8230	JEOL, Tokyo, Japan

## 3. Results and Discussion

### 3.1. Microstructure and Mechanical Properties of Carbon-Deficient WC-10Co Alloy

**Figure 3:**
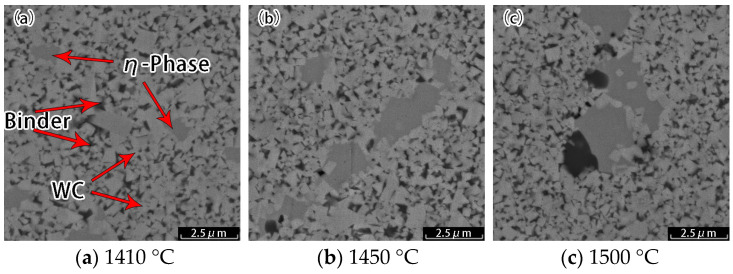
Microstructure of unallocated carbon samples at three sintering temperatures.

**Figure 4:**
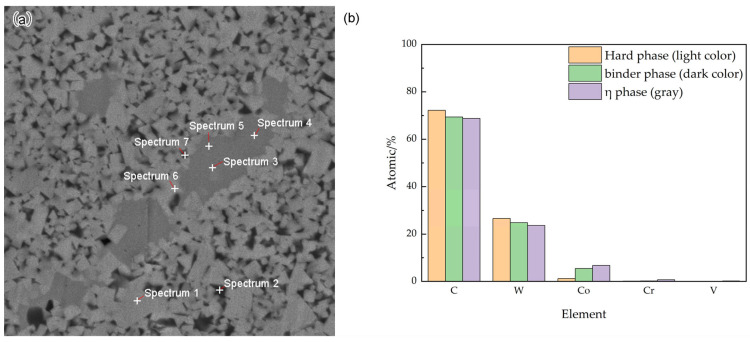
The distribution of various elements in the uncarbonized WC-10Co alloy. (**a**) EDS mapping spot selection for phase analysis, (**b**) Elemental Distribution in Phases.

Figure 3 presents the microstructure photographs of the WC-10Co alloy following sintering at 1410 °C, 1450 °C, and 1500 °C under carbon-deficient conditions. In these images, the light-colored massive grains correspond to the WC hard phase, the dark black regions represent the bonded phase, and the grayish areas in between are identified as the η phase. To analyze the composition of these three phases, we conducted EDS tests at multiple points as illustrated in Figure 4a, with the detection results shown in Figure 4b. EDS analysis indicates a decreasing trend in C and W element contents across the light, dark, and gray phases, respectively, while Co element content exhibits an increasing trend. This distribution aligns with the phase composition characteristics of the hard phase, bonded phase, and η phase.

Under carbon-deficient conditions, a pronounced η brittle phase was observed in the alloy across all sintering temperatures. The formation mechanism of this phase is primarily attributed to carbon deficiency. The carbon content within the WC + γ two-phase region of WC-10Co cemented carbide is restricted to a narrow range, and any deviation from this equilibrium state, particularly a deficiency in carbon, alters the concentrations of W and C in the γ phase. Furthermore, the ultrafine WC powder inherently contains a low total carbon content, coupled with its fine particle size and large specific surface area, which predisposes it to oxygen adsorption during powder mixing and forming. This adsorbed oxygen subsequently reacts with carbon during sintering, exacerbating carbon depletion and promoting the formation of the η phase under carbon-deficient conditions.

In addition, it can be observed from Figure 3 that as the sintering temperature increases, the grain size of WC undergoes notable growth, accompanied by the aggregation and growth of the η phase. Table 4 presents the grain size and mechanical properties of the WC-10Co alloy sintered at different temperatures under carbon-deficient conditions. The average grain size of the carbon-deficient samples ranges from 0.4 to 0.5 μm, exhibiting a positive correlation with the sintering temperature. The mechanical properties of these samples, however, are suboptimal, with the hardness varying from 1630 HV to 1700 HV and decreasing as the sintering temperature rises. Similarly, the flexural strength ranges from 940 MPa to 1300 MPa, also showing a decreasing trend with increasing temperature.

The presence of the η phase leads to inhomogeneous grain growth within the alloy, resulting in the formation of a multi-scale grain organization and laminated structure. This alteration in microstructure has a detrimental effect on the mechanical properties of the alloy, including hardness, wear resistance, and compressive strength. The η phase, being brittle, increases the brittleness of the alloy and reduces its fracture toughness. 

### 3.2. Effect of Carbon Distribution and Sintering Temperature on the Microstructure of WC-10CoNiFe and WC-10Co Alloys

**Figure 5:**
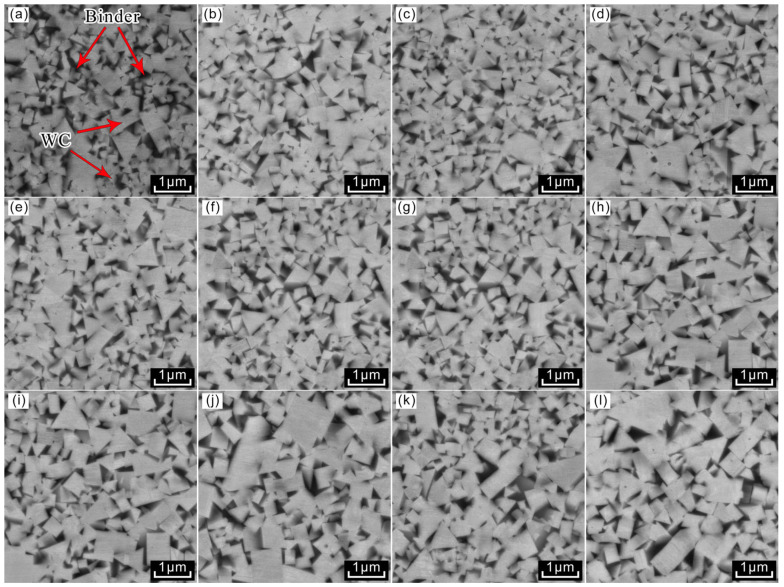
Microstructure of WC-10Co samples in Group A. (**a**) A-1-1410; (**b**) A-2-1410; (**c**) A-3-1410; (**d**) A-4-1410; (**e**) A-1-1450; (**f**) A-2-1450; (**g**) A-3-1450; (**h**) A-4-1450; (**i**) A-1-1500; (**j**) A-2-1500; (**k**) A-3-1500; (**l**) A-4-1500.

**Figure 6:**
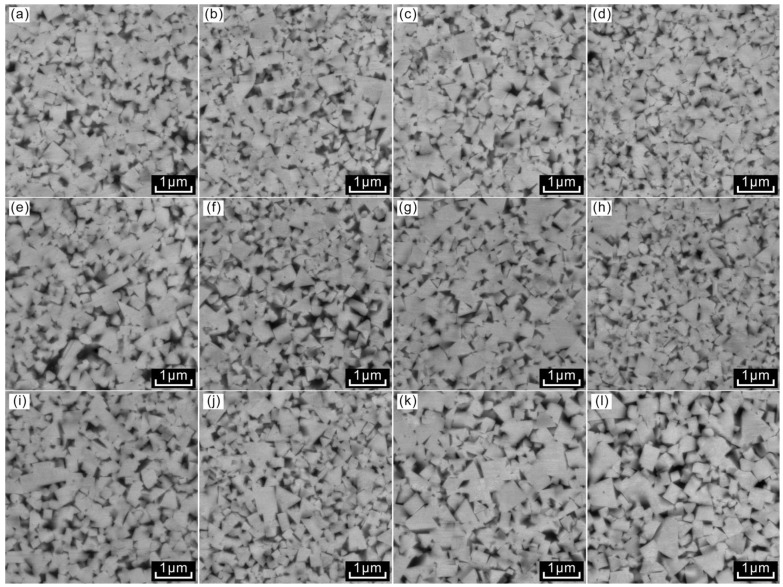
Microstructure of WC-10CoNiFe samples of Group B. (**a**) B-1-1410; (**b**) B-2-1410; (**c**) B-3-1410; (**d**) B-4-1410; (**e**) B-1-1450; (**f**) B-2-1450; (**g**) B-3-1450; (**h**) B-4-1450; (**i**) B-1-1500; (**j**) B-2-1500; (**k**) B-3-1500; (**l**) B-4-1500.

Figure 5 and Figure 6 illustrate the microstructures of WC-10Co samples from Group A and WC-10CoNiFe samples from Group B, sintered at three distinct temperatures with four varying carbon contents of 5.44%, 5.46%, 5.48%, and 5.50%, respectively. Metallographic and SEM observations revealed that, following carbon allocation, neither graphite phase nor brittle η phase emerged in the WC-10Co and WC-10CoNiFe alloys across all four carbon contents. This indicates that the added carbon content was maintained within a reasonable interval.

Cemented carbide primarily comprises two components: the hard phase and the bonded phase. As depicted in Figure 4a, the light-colored, lumpy regions correspond to the hard phase, while the interspersed dark-colored portions represent the bonded phase. Figure 5 and Figure 6 show that the WC grains in both WC-10Co and WC-10CoNiFe alloys predominantly exhibit triangular and polygonal morphologies, with a significant size disparity between the coexisting large and small grains.

The size and morphology of the grains in these alloys demonstrate pronounced changes in response to variations in sintering temperature and carbon content. An increase in sintering temperature leads to a higher proportion of relatively coarse grains in the alloy, thereby elevating the average grain size. Conversely, as carbon content increases, the proportion of fine grains in the alloy initially rises and subsequently decreases.

The pristine WC powder employed in the experiments is nearly spherical, and the morphological transformation of WC grains during sintering can be attributed to the system’s tendency towards minimizing energy and alterations in interfacial tension. Taking the Co-based bonding-phase alloys of Group A as an example, the WC/Co contact interface tends to flatten in the presence of Co. When the sintering temperature reaches the eutectic temperature, the WC grains grow selectively and become more regular in shape under the dissolution–precipitation mechanism, ultimately resulting in the formation of triangular or polygonal grains with a flattened WC/Co interface. This shape does not represent the equilibrium state of WC grains but rather their growth state under the specific preparation conditions of 5.44–5.50% carbon content and sintering temperatures of 1410 °C, 1450 °C, and 1500 °C.

The grain shape of ultrafine-grained cemented carbide is influenced by carbon content, which alters the phase composition within the alloy. When the carbon content is moderate, the phase composition remains stable, enabling the grains to maintain a more regular shape and ensuring a more uniform growth process of the WC grains. However, excessively low carbon content may lead to the appearance of a decarburization phase, while excessively high carbon content may result in the emergence of a carburization phase.

**Figure 7:**
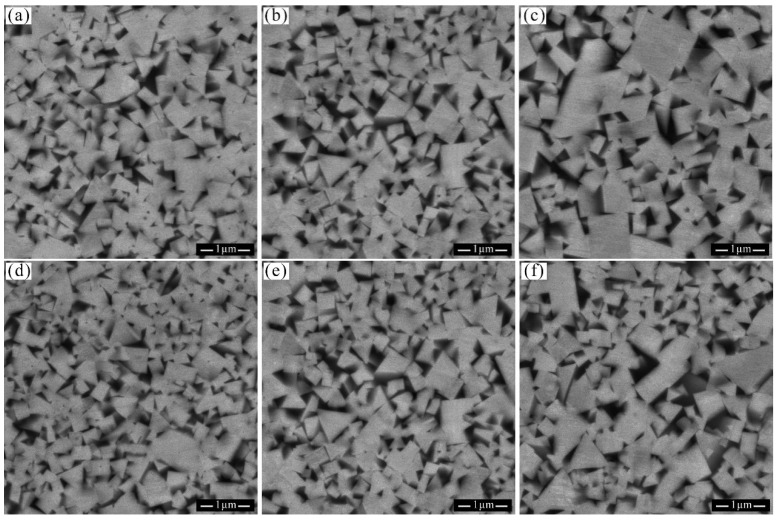
Microstructure of Group A samples with 5.46% and 5.48% carbon content. (**a**) A-2-1410; (**b**) A-2-1450; (**c**) A-2-1500; (**d**) A-3-1410; (**e**) A-3-1450; (**f**) A-3-1500.

Figure 7 presents the microstructures of WC-10Co alloy sintered at different temperatures with carbon contents of 5.46% and 5.48%. It is evident that as the sintering temperature increases, the grain size of the alloy undergoes a certain degree of growth. Notably, a Coarse–Fine grain interwoven structure, comprising grain sizes of 0.14–0.40 μm and 0.51–0.90 μm, emerged when the alloy was sintered at 1450 °C with a carbon content of 5.48%. This Coarse–Fine grain interwoven structure in cemented carbide exhibits a synergistic effect between the coarse and fine grains, where the coarse grains contribute to enhanced plasticity and toughness, while the fine grains provide increased hardness and wear resistance. Consequently, this structure enables the alloy to achieve improved wear resistance while maintaining toughness, resulting in an overall enhancement of its performance. Furthermore, the Coarse–Fine grain interwoven structure effectively restricts dislocation movement, which is beneficial for improving the tensile strength of the material.

**Figure 8:**
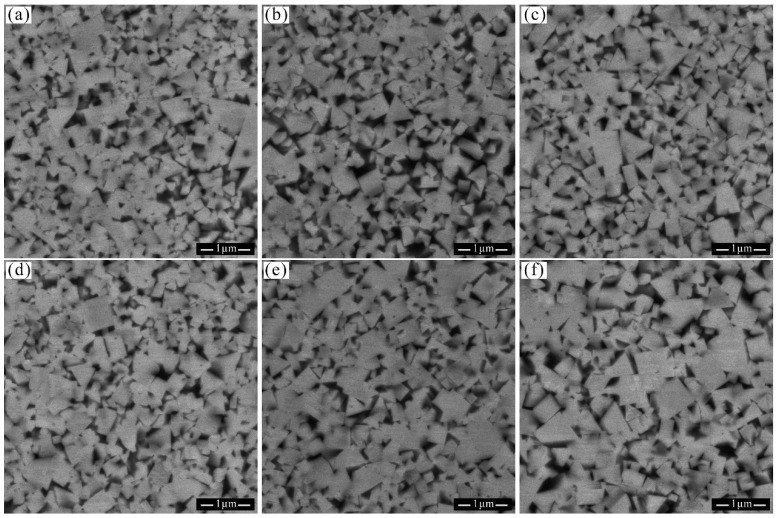
Microstructure of Group B samples with 5.46% and 5.48% carbon content. (**a**) B-2-1410; (**b**) B-2-1450; (**c**) B-2-1500; (**d**) B-3-1410; (**e**) B-3-1450; (**f**) B-3-1500.

Figure 8 illustrate the microstructures of WC-10CoNiFe alloy following sintering at various temperatures with carbon contents of 5.46% and 5.48%. It is apparent that the grain size of the WC-10CoNiFe alloy increases with rising sintering temperature. Additionally, a Coarse–Fine grain interwoven structure, characterized by large grains interspersed with small grains, is observed in the metallographic images.

**Figure 9:**
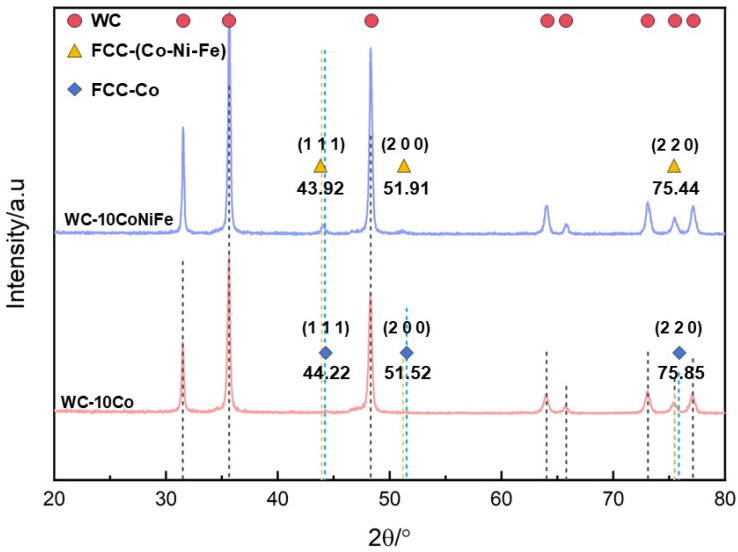
XRD spectra of WC-10CoNiFe and WC-10Co.

X-ray diffraction (XRD) analysis was conducted on WC-10Co and WC-10CoNiFe samples, with phase identification results shown in Figure 9. Comparison with ICDD PDF cards confirmed that both alloys are dominated by the WC hard phase (card no. 73-0471), whose diffraction peaks exhibit significantly higher intensity than the binder phase. The binder phase in WC-10Co is identified as face-centered cubic cobalt (Fcc-Co, card no. 15-0806), while the WC-10CoNiFe alloy forms a (Co-Ni-Fe) solid solution (card no. 12-0736). The dominant peaks of the (Co-Ni-Fe) solid solution correspond to the (111), (200), and (220) crystal planes, consistent with Fcc-Co structure. This suggests that the (Co-Ni-Fe) solid solution retains an Fcc structure. Calculations yield lattice constants of 2.876 Å for Co and 2.880 Å for the (Co-Ni-Fe) solid solution, indicating minimal structural impact from Ni/Fe addition. Notably, the WC diffraction peaks in WC-10CoNiFe are significantly stronger than those in WC-10Co, which correlates with the binder phase’s capacity to solubilize tungsten. Under identical preparation conditions, the synergistic solubilization effect of Ni-Fe in the (Co-Ni-Fe) solid solution effectively suppresses the dissolution–precipitation of WC phase, retaining more tungsten atoms within the hard-phase lattice and thereby enhancing WC peak intensity. No secondary phases such as η-phase or graphite were detected, demonstrating that the experimental design and processing parameters avoided phase imperfections in the alloys.

### 3.3. The Effect of Carbon Distribution and Sintering Temperature on the Grain Size of WC-10CoNiFe and WC-10Co Alloys

The grain size of WC grains exerts a significant influence on the properties of cemented carbide. Generally, as the grain size decreases, the hardness of the cemented carbide increases. However, an excessively small grain size may also lead to a reduction in the material’s toughness. Consequently, during the preparation of ultrafine-grained cemented carbide, it is imperative to comprehensively consider the effects of grain size on both hardness and toughness to achieve cemented carbide with excellent overall performance.

**Figure 10:**
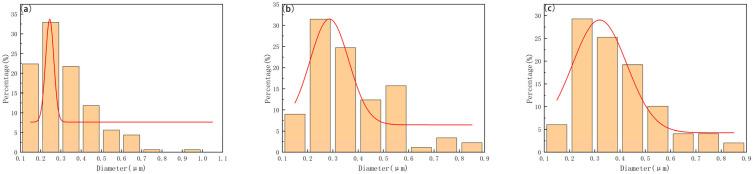
Distribution of grain sizes in WC-10Co samples sintered at different temperatures with gaussian fit 1410 °C (**a**), 1450 °C (**b**), and 1500 °C (**c**).

**Figure 11:**
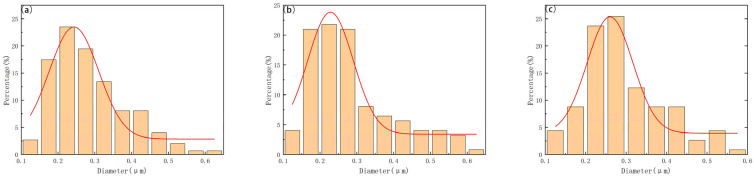
Distribution of grain sizes in WC-10CoNiFe samples sintered at different temperatures with gaussian fit 1410 °C (**a**), 1450 °C (**b**), and 1500 °C (**c**).

**Figure 12:**
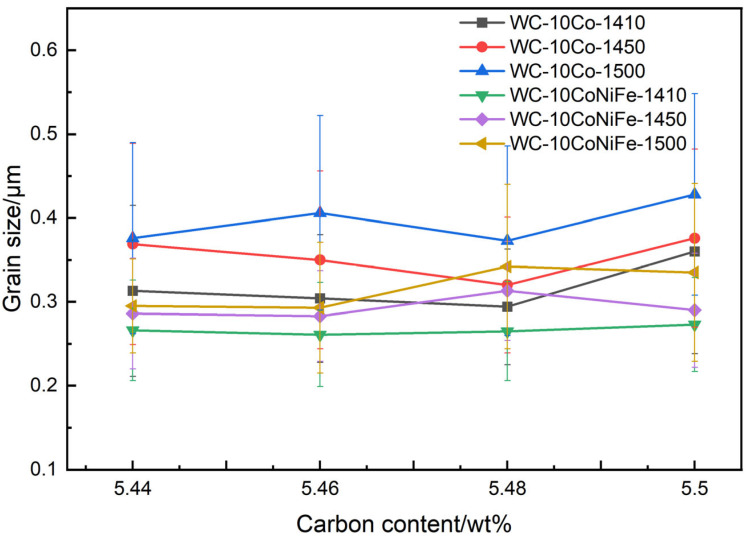
Average particle size of each alloy at different temperatures and carbon contents.

Table 5 presents the grain size data for WC-10Co alloy and WC-10CoNiFe alloy prepared with varying carbon contents and sintering temperatures. Among these, the Group A WC-10Co alloy exhibits the smallest grain size of 0.294 μm at a carbon content of 5.48%, while the Group B WC-10CoNiFe alloy achieves its smallest grain size at a carbon content of 5.46%. Figure 10 and Figure 11 illustrate the effect curves of carbon content and sintering temperature on the grain size of the two alloys. As depicted in Figure 12, the grain size of both alloys demonstrates a trend of decreasing and then increasing with the rise in carbon content. Additionally, the higher the sintering temperature, the larger the grain size of the alloys. Furthermore, upon comparison, it is evident that at the same sintering temperature, WC-10CoNiFe alloy possesses a smaller grain size in comparison to WC-10Co alloy.

The growth of WC grains can be primarily categorized into normal growth and abnormal growth. During the sintering process, the growth of WC grains generally adheres to the Ostwald ripening mechanism, where the size distribution of normally grown grains is uniform, and the rate of grain growth is proportional to the driving force. However, in certain scenarios, the abnormal growth of WC grains may transpire due to factors such as irregular grain morphology and uneven grain boundary migration rates.

The increase in sintering temperature accelerates atomic diffusion during the sintering process. This thermodynamic relationship is mathematically described by the Gibbs-Boltzmann distribution:

(4) $$p=V_{0}e^{\frac{-Es}{KBT}}$$

where *p* represents the diffusion rate; *V*_0_ denotes the vibrational frequency of atoms; *Es* signifies the energy barrier required for diffusion; *KB* is the Boltzmann constant; and *T* corresponds to the absolute temperature.

In other words, the atomic diffusion rate during the sintering stage is proportional to the sintering temperature. As the sintering temperature increases, the atomic migration rate accelerates, thereby expediting grain growth. Consequently, the grain size is also proportional to the sintering temperature.

Generally, as the carbon content increases, the volume of the liquid phase in the sintered body during cemented carbide production augments, and the retention time of the liquid phase is prolonged. This provides enhanced opportunities for the dissolution and precipitation of WC grains in the liquid phase, promoting grain growth. Nevertheless, the experimental results indicate that increasing the carbon content from 5.44% to 5.48% led to a decrease in the average grain size.

In the preceding ball-milling process, the raw material grains were refined, and the ultrafine WC crystals were inherently fine. The dissolved–precipitated WC grains during sintering were even coarser than those prior to sintering. However, the increase in carbon content reduces the solubility of WC in the binder phase and impedes the dissolved–precipitated behavior of the grains within the same sintering duration. Therefore, the increase in carbon content within a certain range results in a decrease in the grain size of the final alloy.

EDS analyses were conducted on Group A WC-10Co and Group B WC-10CoNiFe alloys, as presented in Table 6. The results reveal that elements C and W are concentrated in the hard phase, while elements Co, Ni, Fe, Cr, and V are concentrated in the binder phase. Comparing Groups A and B, with the same total proportion of the hard phase in the alloy, element W in Group B is more concentrated in the hard phase. This suggests that upon completion of sintering, WC is less dissolved in the CoNiFe multi-major-element bonding phase compared to the pure Co bonding phase. Both Groups A and B incorporated VC and Cr_3_C_2_ as inhibitors to suppress grain growth. With the same total proportion of inhibitors in the alloy, Group A exhibits a significantly higher concentration of Cr and V elements in the bonding phase compared to Group B. In contrast, only a relatively small amount of Cr and V elements are present in the hard phase of Group B. This indicates that the inhibitors in Group B are more concentrated at the junction of the hard phase and the bonding phase. The inhibitors hinder the growth of WC grains by adsorbing onto the grain boundaries, resulting in more refined grains in Group B when the preparation process is identical.

### 3.4. The Effect of Carbon Distribution and Sintering Temperature on the Mechanical Properties of WC-10CoNiFe and WC-10Co Alloys

**Figure 13:**
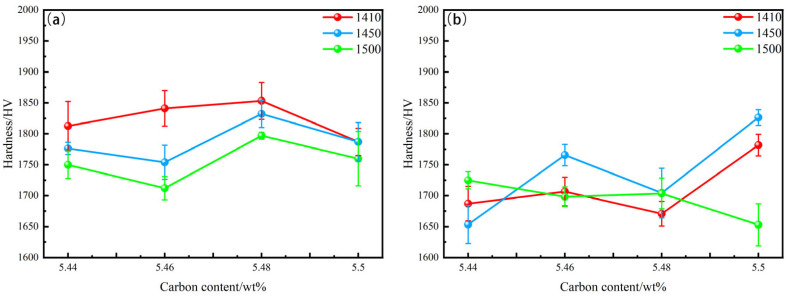
The relationship between microhardness and carbon content in Group A and Group B alloys. (**a**) WC-10Co; (**b**) WC-10CoNiFe.

Figure 13 illustrate the relationship between the microhardness and carbon content of WC-10CoNiFe (Group B) and WC-10Co (Group A) alloys sintered at different temperatures. For Group A alloys, as the sintering temperature increases, the hardness of the alloy exhibits a downward trend. With the increase in carbon content, the hardness of the alloy shows a declining trend. For Group B alloys, the hardness of the samples sintered at 1450 °C is generally higher than that of the samples sintered at the other two temperatures. As the carbon content increases, the hardness of the alloys sintered at 1410 °C and 1450 °C shows a tendency to rise and fall, while the hardness of the alloys sintered at 1500 °C shows a tendency to fall and rise. The hardness of cemented carbide is profoundly influenced by the hard phase. The smaller the size of the WC grain, the higher the hardness of the alloy. This is because grain refinement leads to an increase in the number of grain boundaries and dislocations, which impedes dislocation movement and thereby enhances the hardness of the alloy. As shown in Figure 13a, the lower the sintering temperature, the smaller the grain size, and the higher the hardness. Additionally, when the carbon content in the WC grain appears to be relatively reduced, the hardness of the alloy also increases. In Group B alloys, due to the strong affinity of Fe and Ni for C, these elements are widely present in the bonding phase. During the sintering process, carbon segregation occurs in the WC, and compounds are formed in the bonding phase with Fe and Ni. Higher sintering temperatures undoubtedly exacerbate this behavior. Therefore, the hardness of the alloy sintered at 1450 °C is higher than that of the alloy sintered at 1410 °C in Figure 13b. In contrast, the alloy sintered at 1500 °C has the lowest hardness due to the large particle size. In summary, the hardness of the alloy is determined by the combined effect of grain size and carbon content in the WC grains.

**Figure 14:**
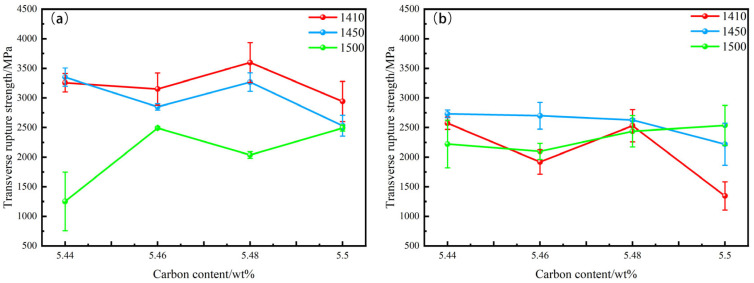
The relationship between transverse rupture strength and carbon content in Group A and Group B alloys. (**a**) WC-10Co; (**b**) WC-10CoNiFe.

As illustrated in Figure 14, the flexural strength of Group A WC-10Co alloy decreases with the increase in sintering temperature. As the carbon content increases, the flexural strength of the alloy sintered at 1410 °C and 1450 °C shows a decreasing trend, while the alloy sintered at 1500 °C exhibits an increasing trend. For Group B WC-10CoNiFe alloy, the flexural strength of the sample sintered at 1450 °C is higher than that of the samples sintered at the other two temperatures. With the increase in carbon content, the flexural strength of the alloy shows a decreasing trend.

The transverse rupture strength of cemented carbide is influenced by several factors, including WC grain size, carbon content, and microphase defects. As the WC grain size decreases, the number of grain boundaries increases, which hinders crack propagation and enhances the flexural strength of the cemented carbide. Therefore, the alloy’s flexural strength increases with the decrease in WC grain size. However, excessive carbon content can lead to the formation of free carbon, while insufficient carbon can result in the η-phase, both of which can reduce the alloy’s strength. The bonding strength between WC grains and the Co bonding phase is stronger than that with Ni or Fe. Thus, substituting Co with Ni or Fe can relatively reduce the alloy’s transverse rupture strength. Internal defects in the alloy can cause stress concentration, accelerating crack formation and reducing the alloy’s strength. Microphase defects are also important factors affecting the transverse rupture strength.

A comprehensive analysis of Figure 13 and Figure 14 reveals that WC-10Co cemented carbide achieves the highest flexural strength of 3598 MPa and hardness of 1853 HV when the carbon content is 5.48% and the sintering temperature is 1410 °C. For WC-10CoNiFe, the optimal overall performance, with a flexural strength of 2999 MPa and hardness of 1765 HV, is obtained when the carbon content is 5.46% and the sintering temperature is 1450 °C.

As shown in Table 7, in terms of transverse rupture strength, the WC-10CoNiFe alloy exhibits a strength of 2999 MPa. Although this value is lower than that of the WC-10Co alloy (3598 MPa), which serves as an important reference in this study, it still demonstrates a significant advantage compared to the 2280 MPa of the WC-8Co NiCu alloy. This indicates that while the WC-10CoNiFe alloy maintains a certain level of transverse rupture strength, it allows room for improvement in other properties, and its strength performance is still significantly superior to some other alloy systems.

In terms of hardness performance, the WC-10CoNiFe alloy reaches 1765 HV. This value is not only significantly higher than the 1508 HV of the WC-9Co alloy but also close to the hardness value of 1783 HV reported for the WC-10Co alloy from another literature source, showcasing its competitiveness in hardness. More remarkably, the WC-10CoNiFe alloy achieves significant grain refinement, with a grain size of only 0.261 μm. This size is significantly smaller than that of the WC-10Co alloy (0.294 μm), the WC-Cr_2_(C,N)-Co alloy (0.445 μm), and the WC-10Co alloy (0.64 μm) reported in another literature source. Grain refinement generally contributes to improving the hardness and strength of alloys. Therefore, the WC-10CoNiFe alloy performs exceptionally well in terms of hardness, and its fine grain structure offers potential for further performance enhancement.

Furthermore, the WC-10Co alloy, serving as a reference object in this study, exhibits excellent performance in both transverse rupture strength and hardness, with values of 3598 MPa and 1853 HV, respectively. These outstanding performances not only highlight the inherent advantages of the WC-10Co alloy but also provide an important reference direction for the performance improvement of the WC-10CoNiFe alloy. By comparing the performances of the WC-10Co alloy and the WC-10CoNiFe alloy, we can more clearly see the advantages of the WC-10CoNiFe alloy in grain refinement, as well as its gap with the WC-10Co alloy in terms of transverse rupture strength and hardness. This comparison not only helps us to deeply understand the performance characteristics of the WC-10CoNiFe alloy but also provides a clear direction for future performance optimization. Specifically, by further optimizing the alloy composition and preparation process, it is expected that the WC-10CoNiFe alloy can further enhance its mechanical properties while maintaining the advantage of grain refinement.

**Figure 15:**
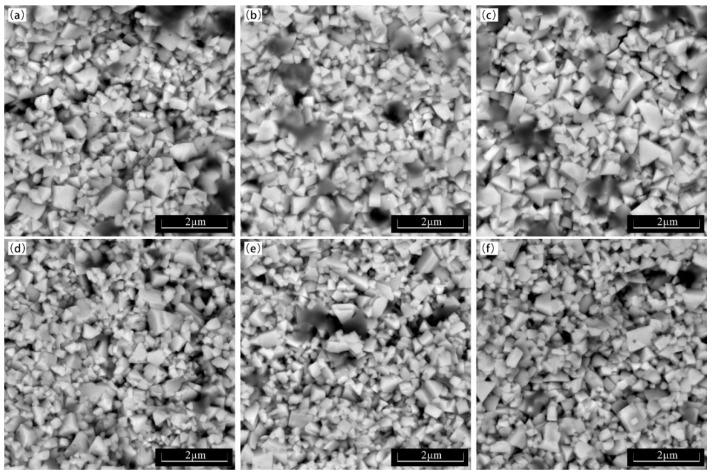
Fracture grain morphology of WC-10Co and WC-10CoNiFe samples for selected preparation conditions. (**a**) A-1-1410; (**b**) A-3-1410; (**c**) A-3-1450; (**d**) B-1-1410; (**e**) B-3-1410; (**f**) B-3-1450.

Not only on the surface but also in the internal grain structure of the alloy, a similar trend is observed. As shown in Figure 15, the polygonal block shape of the grains in the cross-section is essentially the same as that observed under the electron microscope. This indicates that the inherent properties of the grains themselves are not significantly altered during the fracture process. A closer examination of the fracture surfaces reveals that cracks predominantly propagate along the bonding phase, as evidenced by the smooth appearance of the fracture paths. This indicates a strong preference for intergranular fracture, where cracks follow the grain boundaries rather than penetrating through the grains themselves, suggesting that the bonding phase acts as a weak link in the alloy’s fracture behavior.

However, it is also notable that a limited number of relatively larger WC grains exhibit transgranular fracture, where cracks do penetrate through the grains. This phenomenon is particularly evident in regions where the grain size is larger, possibly due to the increased difficulty in deflecting cracks around these larger grains. The rarity of such transgranular fractures further supports the overall brittle nature of these alloys but highlights the importance of grain size distribution in influencing fracture behavior.

To gain a deeper understanding of the fracture mechanism, we examined the influence of carbon content and sintering temperature on the alloy’s properties, as revealed by our experimental data. These parameters, while not substantially altering the inherent properties of the grains, such as their crystallographic structure, significantly affect the morphology and distribution of the bonding phase, thereby influencing the fracture behavior and the final mechanical properties of the alloys.

For instance, in the case of WC-10Co cemented carbides, the alloy reaches its peak performance at a carbon content of 5.48 wt% and a sintering temperature of 1410 °C, exhibiting a maximum transverse rupture strength of 3598 MPa and a hardness of 1853 HV, with a grain size of 0.294 µm. This excellent mechanical performance is closely related to the fine grain size and uniform distribution within the alloy. As the sintering temperature increases or the carbon content deviates from this optimum, both the transverse rupture strength and hardness of the alloy decrease. This suggests that the increase in grain size and the alteration in bonding-phase properties may facilitate crack propagation along the bonding phase, thereby reducing the alloy’s overall performance.

Similarly, for WC-10CoNiFe cemented carbides, our experimental data also demonstrates the significant impact of carbon content and sintering temperature on the alloy’s properties. The alloy exhibits its optimal mechanical performance at a carbon content of 5.46 wt% and a sintering temperature of 1450 °C, with a transverse rupture strength of 2999 MPa, a hardness of 1765 HV, and a grain size of 0.261 µm. Compared to WC-10Co alloys, WC-10CoNiFe alloys demonstrate a finer grain size under optimal conditions, primarily due to the introduction of the Co-Ni-Fe multi-principal-element binder phase, which effectively inhibits grain growth.

The difference in binder phases between Group A and Group B alloys, while not leading to significant structural differences in the overall grain morphology, results in variations in their mechanical properties and microstructures, particularly in terms of grain size and hardness. This further underscore the crucial role of the binder phase’s characteristics, including its composition, distribution, and interaction with WC grains, in determining the final properties of the alloys.

In conclusion, by carefully controlling the carbon content and sintering temperature, we can significantly optimize the microstructure and mechanical properties of ultrafine-grained WC-10CoNiFe and WC-10Co cemented carbides. The experimental data provides valuable insights for a deeper understanding of the fracture behavior of these alloys and offers guidance for the development of new cemented carbide materials with superior performance.

## 4. Conclusions

In this study, the pivotal roles of carbon content and sintering temperature in governing the microstructure and mechanical properties of ultrafine tungsten carbide-based cemented carbides were systematically examined. The experimental results demonstrate that elevating the carbon content within the range of 5.44–5.50 wt% effectively suppresses the formation of the η-phase and refines the WC grains, while an increase in the sintering temperature (1410–1500 °C) facilitates grain coarsening by enhancing atomic diffusion. Under optimal conditions (5.46 wt% carbon content, sintering temperature 1450 °C), the finer grain size (0.261 µm) of the WC-10CoNiFe alloy, as compared to the WC-10Co alloy (0.294 µm), is ascribed to the influence of the multi-primary-element binder on the dissolution–precipitation behavior of WC.

Mechanical property evaluations reveal that the conventional WC-10Co system exhibits superior properties, with a hardness of 1853 HV and a flexural strength of 3898 MPa, whereas the WC-10CoNiFe alloy maintains a satisfactory hardness (1826 HV) despite a reduced flexural strength of 3031 MPa. This disparity underscores the inherent advantages of cobalt in terms of stress transfer efficiency and resistance to crack propagation and also indicates that there is potential for further enhancement in the design of the bonding phase.

A critical process window (5.46–5.48 wt% carbon content, sintering temperature 1450 °C) was established, which eliminates the formation of the brittle η phase while optimizing grain refinement. These findings offer a valuable reference for tailoring ultrafine-grained multi-major-element bonded-phase cemented carbides through precise carbon content–temperature coordination.

## Figures and Tables

**Table 1:** The technical parameters of the experimental raw materials.

Materials	WC	Co	Ni	Fe	C	Cr_3_C_2_	VC
Particle/μm	0.4	1.33	2.0	3.08	11.8	2.59	1.94
Oxygen Content/wt%	0.16	0.21	0.11	0.18	0.19	0.23	0.12

**Table 2:** Composition of groups of cemented carbide.

Samples	Composition/wt%						
	WC	Co	Ni	Fe	C	Cr_3_C_2_	VC
A-1	89.22	10.00	0	0	0.08	0.50	0.20
A-2	89.20	10.00	0	0	0.10	0.50	0.20
A-3	89.18	10.00	0	0	0.12	0.50	0.20
A-4	89.17	10.00	0	0	0.13	0.50	0.20
B-1	89.22	5.31	4.17	0.52	0.08	0.50	0.20
B-2	89.20	5.31	4.17	0.52	0.10	0.50	0.20
B-3	89.18	5.31	4.17	0.52	0.12	0.50	0.20
B-4	89.17	5.31	4.17	0.52	0.13	0.50	0.20

**Table 4:** Grain size and mechanical properties of carbon-deficient WC-10Co alloys.

Sintering Temperature/°C	Grain Size/μm	Transverse Rupture Strength/MPa	Hardness/HV
1410	0.43	1300	1700
1450	0.45	1220	1680
1500	0.47	940	1630

**Table 5:** Grain size of alloy samples.

Sintering Temperature of Group A/°C	Carbon Content/wt%	Grain Size/μm	Sintering Temperature of Group B/°C	Carbon Content/wt%	Grain Size/μm
1410	5.44	0.313	1410	5.44	0.266
1410	5.46	0.304	1410	5.46	0.261
1410	5.48	0.294	1410	5.48	0.265
1410	5.50	0.360	1410	5.50	0.273
1450	5.44	0.369	1450	5.44	0.286
1450	5.46	0.350	1450	5.46	0.283
1450	5.48	0.320	1450	5.48	0.313
1450	5.50	0.376	1450	5.50	0.290
1500	5.44	0.376	1500	5.44	0.295
1500	5.46	0.406	1500	5.46	0.293
1500	5.48	0.373	1500	5.48	0.342
1500	5.50	0.428	1500	5.50	0.335

**Table 6:** The distribution of elements in the two phases of the alloy.

Elements in WC-10Co	Hard Phase Atomic/%	Binder Phase Atomic/%	Elements in WC-10CoNiFe	Hard Phase Atomic/%	Binder Phase Atomic/%
C	52.91	29.52	C	31.49	23.73
V	0.24	2.42	V	0.05	0.68
Cr	0.32	3.18	Cr	0.47	1.55
Fe			Fe	0.68	1.73
Co	5.41	32.66	Co	5.20	17.08
Ni			Ni	4.33	14.13
W	41.14	32.21	W	57.78	41.1

**Table 7:** Performance comparison of WC-10Co and WC-10CoNiFe [25,26,27,28].

Alloys	Transverse Rupture Strength/MPa	Harness	Grain Size/μm
WC-10Co	3598	1853 HV	0.294
WC-10CoNiFe	2999	1765 HV	0.261
WC-Cr_2_(C,N)-Co [25]		1842 HV	0.445
WC-8Co NiCu [26]	2280	87.7 HRA	
WC-9Co [27]	3514	1508 HV	
WC-10Co [28]	3600	1783 HV	0.64

## Data Availability

The original contributions presented in this study are included in the article. Further inquiries can be directed to the corresponding authors.
